# Concomitant antihypertensive medication and outcome of patients with metastatic castration‐resistant prostate cancer receiving enzalutamide or abiraterone acetate

**DOI:** 10.1002/cam4.6853

**Published:** 2024-01-02

**Authors:** Ondřej Fiala, Petr Hošek, Hana Korunková, Milan Hora, Jiří Kolář, Ondřej Šorejs, Ondřej Topolčan, Jan Filipovský, Václav Liška, Matteo Santoni, Sebastiano Buti, Jindřich Fínek

**Affiliations:** ^1^ Department of Oncology and Radiotherapeutics, Faculty of Medicine and University Hospital in Pilsen Charles University Pilsen Czech Republic; ^2^ Biomedical Center, Faculty of Medicine in Pilsen Charles University Pilsen Czech Republic; ^3^ Department of Urology, Faculty of Medicine and University Hospital in Pilsen Charles University Pilsen Czech Republic; ^4^ Department of Immunochemistry Diagnostics, University Hospital in Pilsen Charles University Pilsen Czech Republic; ^5^ 2nd Department of Internal Medicine, Faculty of Medicine and University Hospital in Pilsen Charles University Pilsen Czech Republic; ^6^ Department of Surgery, University Hospital in Pilsen Charles University Pilsen Czech Republic; ^7^ Oncology Unit Macerata Hospital Macerata Italy; ^8^ Department of Medicine and Surgery University Hospital of Parma Parma Italy; ^9^ Oncology Unit University Hospital of Parma Parma Italy

**Keywords:** abiraterone acetate, angiotensin‐converting enzyme inhibitors, antihypertensives, castration‐resistant prostate cancer, comedication, enzalutamide, novel hormonal therapies

## Abstract

**Background:**

The introduction of novel hormonal therapies represented by enzalutamide (ENZ) and abiraterone acetate (ABI) has reached a great progress in the treatment of metastatic castration‐resistant prostate cancer (mCRPC). The majority of mCRPC patients are elderly suffering from chronic co‐morbidities requiring use of various concomitant medications. In the present study, we focused on impact of concomitant antihypertensive medication on the outcomes of mCRPC patients treated with ENZ or ABI.

**Methods:**

In total, 300 patients were included and their clinical data were retrospectively analyzed.

**Results:**

Angiotensin‐converting enzyme inhibitors (ACEIs) represented the only concomitant medication significantly associated with survival. The median radiographic progression‐free survival (rPFS) and overall survival (OS) for patients using ACEIs were 15.5 and 32.3 months compared to 10.7 and 24.0 months for those not using ACEIs (*p* = 0.0053 and *p* = 0.0238, respectively). Cox multivariable analysis revealed the use of ACEIs a significant predictive factor for both rPFS (HR = 0.704, *p* = 0.0364) and OS (HR = 0.592, *p* = 0.0185).

**Conclusion:**

The findings of this study suggest an association between the concomitant use of ACEIs and longer survival of mCRPC patients receiving ENZ or ABI therapy.

## Graphical abstract

Our results suggest that there is a significant association between the use of angiotensin‐converting enzyme inhibitors (ACEIs) and favorable outcome of patients with metastatic castration‐resistant prostate cancer (mCRPC) treated with enzalutamide or abiraterone acetate. Our study also indirectly indicates that ACEIs might be a preferred type of antihypertensive drugs for these patients with mCRPC.

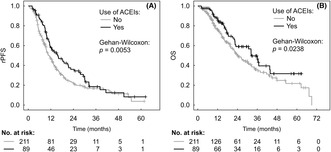

## 1 BACKGROUND

Prostate cancer (PC) represents most frequently diagnosed malignancy in men worldwide.^1^, ^2^ There is a substantial proportion of metastatic PC patients, who develop metastatic castration‐resistant PC (mCRPC), which was defined as castrate testosterone levels and a radiographic progression and/or biochemical progression of prostate‐specific antigen (PSA) levels on androgene deprivation therapy (ADT).^3^ In the last decade, a great progress has been reached in the systemic therapy for mCRPC patients. The introduction of novel hormonal therapies (NHTs) represented by enzalutamide (ENZ), abiraterone acetate (ABI), apalutamide and darolutamide has led to a substantial improvement of patient survival.^4^, ^5^, ^6^, ^7^, ^8^, ^9^ Despite this indisputable therapeutic revolution, the prognosis of mCRPC remains poor and further treatment optimization seems to be desirable.

The majority of mCRPC patients are elderly suffering from various chronic co‐morbidities requiring use of several concomitant medications. Cardiovascular diseases in particular represent the most common group of chronic co‐morbidities in the PC patient population.^10^, ^11^ The emerging issue that should be elucidated is whether the concomitant administration of cardiovascular medication may affect the efficacy of anticancer therapy in these patients. Even though several reports previously suggested that specific antihypertensive agents may be associated with the outcomes of PC patients, the results are controversial.^12^, ^13^, ^14^, ^15^, ^16^, ^17^, ^18^, ^19^, ^20^, ^21^, ^22^, ^23^, ^24^ Notably, the data on mCRPC are very limited and this field remains unexplored.

In our retrospective study we focused on the possible impact of concomitant exposure to common antihypertensive medication on the outcomes of mCRPC patients receiving ENZ or ABI.

## 2 PATIENTS AND METHODS

### 2.1 Study design

Data from mCRPC patients receiving ENZ or ABI were analyzed. We retrospectively assessed the association between radiographic progression‐free survival (rPFS) and overall survival (OS) and the use of antihypertensive medications including beta‐blockers (BBs), angiotensin‐converting enzyme inhibitors (ACEIs), angiotensin II receptor blockers (ARBs), calcium channel blockers (CCBs) and diuretics. Clinical data were extracted from the hospital information system. The use of concomitant antihypertensive medication was assessed at the beginning of ENZ or ABI therapy. All the assessed concomitant medications were administered individually.

The protocol of the study and the form of Informed consent for participants were approved by the Ethical Committee of the Faculty of Medicine and University Hospital in Pilsen on June 7, 2021 (No. 245/2021) and complied with the International Ethical Guidelines for Biomedical Research, the Declaration of Helsinki, and local laws. The Informed consent was obtained from all the participants.

### 2.2 Patients and treatment

Patients with histologically confirmed mCRPC fulfilling the criteria for castration resistance were treated with ENZ or ABI between 2007 and 2022 at the Department of Oncology and Radiotherapeutics, University Hospital in Pilsen, Pilsen, Czech Republic. ENZ (Xtandi, Astellas Pharma Inc., Tokyo, Japan) was administered orally in the standard approved dose (160 mg daily). ABI (Zytiga, Janssen Pharmaceuticals Co., Beerse, Belgium) was administered orally in the standard approved schedule (1000 mg daily) in combination with prednisone (10 mg daily). The therapy with ENZ or ABI was continued until disease progression, unacceptable toxicity, or patient refusal.

The routine clinical checks including physical examination and biochemical laboratory tests with PSA were performed each moth, and radiographic controls using computed tomography (CT) or positron emission tomography‐CT (PET/CT) or PET‐magnetic resonance (PET/MR) were performed every 3 to 6 months. The objective response to therapy was assessed according to the Response Evaluation Criteria in Solid Tumors (RECIST).^25^

### 2.3 Statistical analysis

Standard descriptive statistics and frequencies were used to characterize the sample data set. Baseline clinical characteristics of antihypertensive users and nonusers were compared using the *t*‐test (normally distributed continuous variables), the Mann–Whitney *U*‐test (non‐normally distributed continuous variables), Fisher's exact test and the chi‐squared test (categorical variables). Overall survival (OS) was defined as a time from the date of treatment initiation until the date of death. Radiographic progression‐free survival (rPFS) was defined as a time from the date of treatment initiation until the date of first documented radiographic progression or death. Patients in whom the terminal event had not occurred were censored at the date of the last follow‐up. OS and rPFS were estimated using the Kaplan–Meier method and point estimates were accompanied by two‐sided 95% confidence intervals. The Gehan‐Wilcoxon test was used for the assessment of statistical significance of the differences in survival between users and nonusers of individual comedication types. Multivariable Cox proportional hazards model was then used to verify the prognostic independence of antihypertensive usage of other common clinical factors. The median follow‐up time was estimated using the inverse the Kaplan–Meier method. The level of statistical significance was set at *α* = 0.05 and all reported *p*‐values are two‐tailed. The statistical analysis was performed using STATISTICA (Version 12; StatSoft, Inc., TuIsa, OK, USA).

## 3 RESULTS

### 3.1 Patient characteristics

In total, 300 mCRPC patients were included in our study and their characteristics are summarized in Table 1.

**TABLE 1:** Baseline patient characteristics.

Characteristic, *n* (%)	All patients (*n* = 300)	ACEI users (*n* = 89)	ACEI nonusers (*n* = 211)	*p* value* (test)
Age at treatment initiation (years)				0.7444 (*t*‐test)
Median (range)	72.9 (51.5–87.5)	73.3 (51.9–84.1)	72.1 (51.5–87.5)	
PSA at treatment initiation				0.8148 (Mann–Whitney *U*)
Median (range)	24.9 (0–2006)	24.2 (0–1540)	26.6 (0–2006)	
Gleason score				0.9231 (chi‐square)
Three–six	44 (16.0%)	12 (15.2%)	32 (16.3%)	
Seven	83 (30.2%)	23 (29.1%)	60 (30.6%)	
Eight–ten	148 (53.8%)	44 (55.7%)	104 (53.1%)	
Therapy				0.9147 (chi‐square)
Enzalutamide	157 (52.3%)	47 (52.8%)	110 (52.1%)	
Abiraterone acetate	143 47.7%)	42 (47.2%)	101 (47.9%)	
Synchronous metastases				0.6549 (chi‐square)
Yes	139 (46.3%)	43 (48.3%)	96 (45.5%)	
No	161 (53.7%)	46 (51.7%)	115 (54.5%)	
Previous docetaxel				0.6794 (chi‐square)
No	234 (78.3%)	71 (79.8%)	163 (77.6%)	
Yes	65 (21.7%)	18 (20.2%)	47 (22.4%)	
Previous prostatectomy				0.4800 (chi‐square)
Yes	58 (19.3%)	15 (16.9%)	43 (20.4%)	
No	242 (80.7%)	74 (83.1%)	168 (79.6%)	
Previous radiotherapy				0.2757 (chi‐square)
Yes	129 (43.0%)	34 (38.2%)	95 (45.0%)	
No	171 (57.0%)	55 (61.8%)	116 (55.0%)	
Metastatic sites				0.0516 (chi‐square)
Lymph nodes	34 (11.4%)	12 (13.6%)	22 (10.4%)	
Bone	218 (72.9%)	56 (63.6%)	162 (76.8%)	
Visceral	47 (15.7%)	20 (22.7%)	27 (12.8%)	
Subsequent therapy				0.5873 (Fisher)
Yes	94 (31.3%)	30 (33.7%)	64 (30.3%)	
No	206 (68.7%)	59 (66.3%)	147 (69.7%)	
Number of subsequent therapy lines				0.9745 (Mann–Whitney *U*)
None	226 (75.3%)	67 (75.3%)	159 (75.4%)	
One	55 (18.3%)	16 (18.0%)	39 (18.5%)	
Two	18 (6.0%)	6 (6.7%)	12 (5.7%)	
Three	0	0	0	
Four	1 (0.3%)	0	1 (0.5%)	
Type of subsequent therapy				1 (Fisher)
Docetaxel	41 (13.7%)	16 (18.0%)	25 (11.8%)	
Cabazitaxel	16 (5.3%)	4 (4.5%)	12 (5.7%)	
Enzalutamide or abiraterone acetate	29 (9.7%)	6 (6.7%)	23 (10.9%)	
Radium 223	9 (3.0%)	2 (2.2%)	7 (3.3%)	

Abbreviations: ACEI, angiotensin‐converting enzyme inhibitor; PSA, prostate‐specific antigen.

* *p*‐Value for comparison between ACEI users and nonusers.

At the time of ENZ or ABI initiation 81 (27.0%) were using BBs, 89 (29.7%) were using ACEIs, 59 (19.7%) were using ARBs, 84 (28.0%) were using CCBs, and 71 (23.7%) were using diuretics. Also, 22 (7.3%) patients were using other antihypertensive medication, always in combination with at least one of the previous types. In total, 189 patients (63%) were using some type of antihypertensive medication.

### 3.2 Patient survival

Median rPFS and OS for the whole cohort were 12.1 months (95% CI 10.6–13.7) and 26.7 months (95% CI 22.5–32.5), respectively. At the time of data analysis 214 (71.3%) patients progressed, 142 (47.3%) patients died and the median follow‐up time was 29.3 months.

**FIGURE 1:**
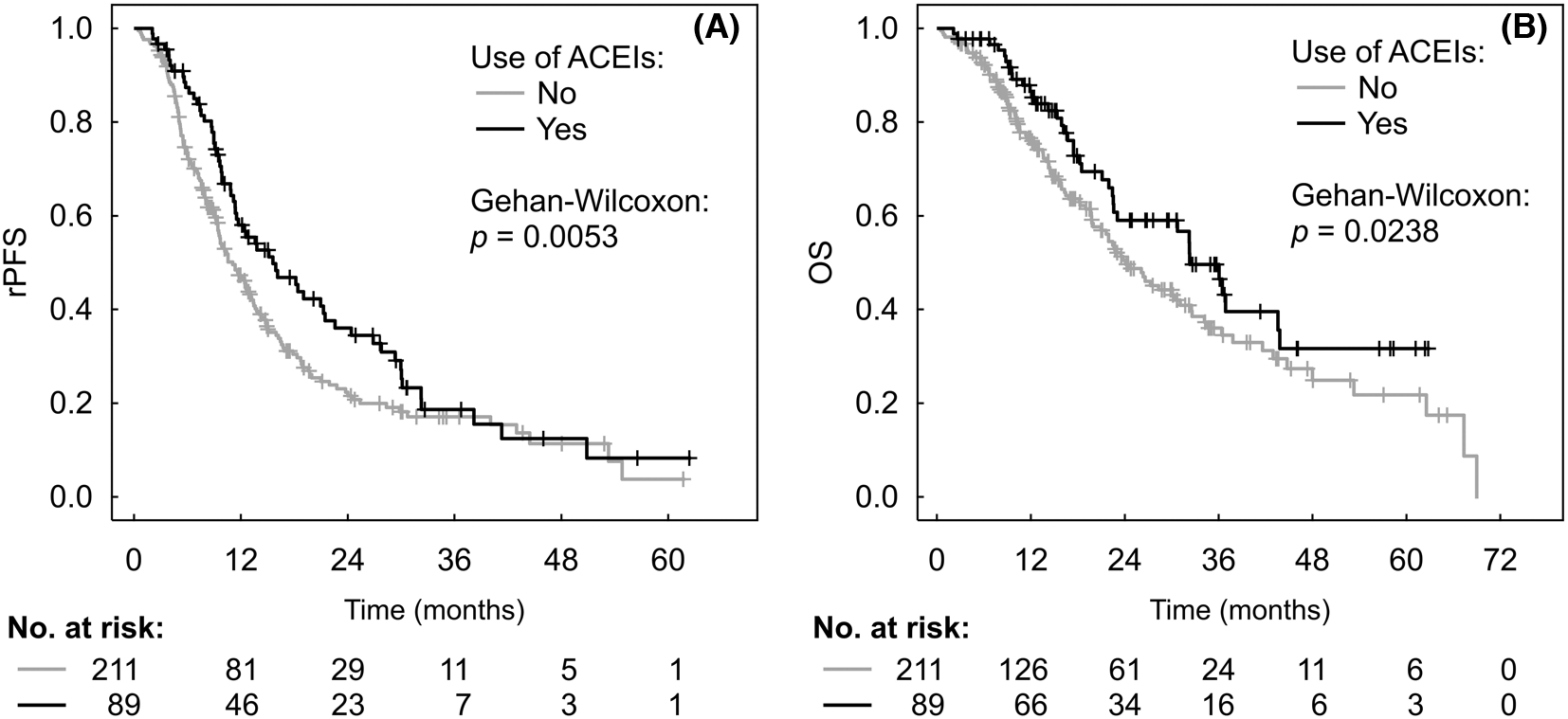
Kaplan–Meier estimates of radiographic progression‐free survival (rPFS) (A) and overall survival (OS) (B) according to the use of angiotensin‐converting enzyme inhibitors (ACEIs).

The median rPFS and OS for patients with arterial hypertension treated with any antihypertensive medication were 13.6 (95% CI 11.4–16.5) and 30.1 (95% CI 22.6–36.7) months compared to 9.4 (95% CI 7.8–12.2) and 22.5 (95% CI 18.7–32.6) months for those without arterial hypertension (*p* = 0.0029 and *p* = 0.1104, respectively) (Table 2; Figure 1S).

**TABLE 2:** The Kaplan–Meier analysis with Gehan‐Wilcoxon test assessing the impact of the used concomitant medications on radiographic progression‐free survival (rPFS) and overall survival (OS).

Medication	rPFS		OS	
	Median survival months, (95% CI)	*p*‐Value	Median survival months, (95% CI)	*p*‐Value
Any antihypertensive medication		**0.0029**		0.1104
Nonusers	9.4 (7.8–12.2)		22.5 (18.7–32.6)	
Users	13.6 (11.4–16.5)		30.1 (22.6–36.7)	
Beta‐blockers (BBs)		0.1318		0.8982
Nonusers	11.4 (9.8–13.7)		27.9 (21.9–33.8)	
Users	13.0 (10.1–20.1)		25.0 (19.3–39.3)	
Angiotensin‐converting enzyme inhibitors (ACEIs)		**0.0053**		**0.0238**
Nonusers	10.7 (9.4–13.0)		24.0 (20.0–30.6)	
Users	15.5 (11.4–21.3)		32.3 (22.6–43.6)	
Angiotensin II receptor blockers (ARBs)		0.6912		0.8659
Nonusers	11.7 (10.1–13.4)		26.1 (21.5–32.5)	
Users	13.8 (9.6–19.4)		30.1 (21.9–50.2)	
Renin‐angiotensin system inhibitors (RASIs) = ACEIs or ARBs		**0.0089**		**0.0492**
Nonusers	10.4 (9.1–12.6)		22.6 (19.3–30.9)	
Users	14.5 (11.4–18.9)		32.3 (23.0–43.6)	
Calcium channel blockers (CCBs)		0.2714		0.5372
Nonusers	11.5 (9.6–13.4)		23.8 (21.8–32.6)	
Users	13.4 (11.0–21.0)		30.6 (21.8–53.4)	
Diuretics		0.3767		0.5317
Nonusers	11.5 (10.0–13.6)		26.4 (22.0–32.3)	
Users	13.4 (9.5–22.1)		29.3 (18.5–49.6)	

Statistically significant values are in bold.

The Kaplan–Meier analysis with Gehan‐Wilcoxon test assessing the impact of concomitant antihypertensive medication on patients' survival found that ACEIs were the only individual medication type with significant impact on rPFS and OS, increasing the observed median survival times by 45% (*p* = 0.0053) and 35% (*p* = 0.0238), respectively (Table 2).

The median rPFS and OS for patients using ACEIs were 15.5 (95% CI 11.4–21.3) and 32.3 (95% CI 22.6–43.6) months compared to 10.7 (95% CI 9.4–13.0) and 24.0 (95% CI 20.0–30.6) months for those not using ACEIs (*p* = 0.0053 and *p* = 0.0238, respectively) (Table 3, Figure 1).

**TABLE 3:** Radiographic progression‐free (rPFS) and OS data according to the use of ACEIs.

Survival	Use of ACEIs		*p*‐Value
	No	Yes	
rPFS			**0.0053** (Gehan‐Wilcoxon)
Median (months)	10.7 (9.4–13.0)	15.5 (11.4–21.3)	
6 months	72.5% (66.4–78.7)	86.7% (79.6–93.9)	
12 months	46.7% (39.6–53.8)	57.5% (46.8–68.2)	
24 months	21.6% (15.1–28.1)	34.8% (23.8–45.8)	
OS			**0.0238** (Gehan‐Wilcoxon)
Median (months)	24.0 (20.0–30.6)	32.3 (22.6–43.6)	
12 months	76.1% (69.9–82.2)	86.0% (78.4–93.5)	
24 months	49.9% (41.9–58.0)	58.7% (46.6–70.8)	
36 months	34.9% (26.1–43.6)	46.5% (32.8–60.2)	

Abbreviations: ACEIs, angiotensin‐converting enzyme inhibitors; rPFS, radiographic progression‐free; OS, overall survival.

Statistically significant values are in bold.

The results of the Cox multivariable analysis show that the use of ACEIs remains a significant factor associated with both rPFS (HR = 0.704 [95% CI 0.506–0.978], *p* = 0.0364) and OS (HR = 0.592 [95% CI 0.383–0.916], *p* = 0.0185) (Table 4). Other independent favorable factors for superior rPFS were pre‐chemotherapy setting of ENZ or ABI treatment and metachronous metastatic disease, while other independent favorable factors for superior OS included Gleason score of 7 or lower, metachronous metastatic disease, and the absence of visceral metastases.

**TABLE 4:** Multivariable Cox proportional hazards model for radiographic progression‐free (rPFS) and overall survival (OS).

Parameter, category	rPFS		OS	
	Hazard ratio (95% CI)	*p*‐Value	Hazard ratio (95% CI)	*p* Value
Age at treatment initiation		0.4956		0.6839
Per 1 year increase	0.993 (0.972–1.014)		1.006 (0.979–1.033)	
PSA at treatment initiation		0.5516		0.0993
Per 100 ng/mL increase	1.015 (0.967–1.064)		1.045 (0.992–1.100)	
Gleason score		0.1335		**0.0338**
≤7	1		1	
>7	1.249 (0.934–1.671)		1.491 (1.031–2.157)	
Metastases		**0.0151**		**0.0028**
Metachronous	1		1	
Synchronous	1.451 (1.075–1.960)		1.801 (1.225–2.648)	
Visceral metastases		0.0906		**0.0009**
Absent	1		1	
Present	1.424 (0.946–2.145)		2.279 (1.402–3.706)	
Previous docetaxel		**0.0477**		0.3590
No	1		1	
Yes	1.430 (1.004–2.038)		1.226 (0.794–1.893)	
Use of ACEIs		**0.0364**		**0.0185**
No	1		1	
Yes	0.704 (0.506–0.978)		0.592 (0.383–0.916)	

Abbreviations: ACEIs, angiotensin‐converting enzyme inhibitors; CI, confidence interval; HR, hazard ratio; OS, overall survival; PSA, prostate‐specific antigen; rPFS, radiographic progression‐free.

Statistically significant values are in bold.

Additionally, the effects of ACEIs, ARBs, or RASIs were analyzed for ABI or ENZ users separately. Among patients treated with ABI; the median rPFS and OS for ACEIs users were 21.1 (95% CI 11.5–28.9) and 31.0 (not available) months compared to 11.7 (95% CI 10.1–14.7) and 22.7 (95% CI 18.8–33.8) months for those not using ACEIs (*p* = 0.0728 and *p* = 0.0692, respectively); the median rPFS and OS for patients using ARBs were 14.8 (95% CI 10.1–29.3) and 31.9 (not available) months compared to 12.7 (95% CI 10.6–16.2) and 22.8 (95% CI 19.8–32.8) months for those not using ARBs (*p* = 0.4879 and *p* = 0.3547, respectively); the median rPFS and OS for patients using RASIs (ACEIs or ARBs) were 19.0 (95% CI 11.5–23.4) and 31.5 (not available) months compared to 11.4 (95% CI 9.7–14.4) and 22.2 (95% CI 18.2–31.4) months for those not using RASIs (*p* = 0.0661 and *p* = 0.0378, respectively). Among patients treated with ENZ; the median rPFS and OS for patients using ACEIs were 12.5 (95% CI 9.8–17.1) and 33.1 (95% CI 20.1–40.7) months compared to 9.4 (95% CI 7.8–12.7) and 24.6 (95% CI 19.7–32.6) months for those not using ACEIs (*p* = 0.0224 and *p* = 0.1705, respectively); the median rPFS and OS for patients using ARBs were 10.6 (95% CI 7.8–20.6) and 27.5 (95% CI 15.8–44.4) months compared to 10.0 (95% CI 9.1–12.7) and 27.1 (95% CI 19.7–36.2) months for those not using ARBs (*p* = 0.8039 and *p* = 0.7780, respectively); the median rPFS and OS for patients using RASIs (ACEIs or ARBs) were 12.6 (95% CI 9.7–17.9) and 31.0 (95% CI 22.7–38.4) months compared to 9.0 (95% CI 6.9–12.6) and 23.1 (95% CI 15.9–33.7) months for those not using RASIs (*p* = 0.0269 and *p* = 0.3766, respectively) (Table S1).

## 4 DISCUSSION

The advent of NHTs brought a great progress into the systemic treatment of mCRPC and recently, also into the treatment of metastatic hormone‐sensitive PC (mHSPC). The data from our retrospective study suggest that the concomitant use of ACEIs is significantly associated with better outcome in mCRPC patients receiving ENZ or ABI. Among all the investigated antihypertensive medications, only the use of ACEIs showed significant association with patients' outcome. Furthermore, the results of the multivariate Cox proportional hazards model revealed the concomitant use of ACEIs as an independent predictive factor for both rPFS and OS.

The circulating renin‐angiotensin system (RAS) is fundamentally known to regulate blood pressure and electrolyte homeostasis. In addition, local RAS acting at the cellular level is involved in various important biological processes including cell growth, metabolism and proliferation.^26^ It is expressed in many tissues including cancer cells and tumor microenvironment.^27^, ^28^ The local RAS has been proposed to have an important role in development and pathophysiology of PC.^29^ Two RAS signaling pathways can be distinguished, classical and alternative, both involved in cancer‐related processes. The classical RAS signaling is mainly represented by the Angiotensin (Ang) II/AT1 and AT2 receptor (AT1R, AT2R) pathway. Besides its major role in the constitution of cardiovascular homeostasis, it can stimulate angiogenesis, inflammation and also cancer cell proliferation acting thru the activation of mitogen‐activated protein kinase (MAPK) and signal transducer activator of transcription 3 (STAT3) signaling pathways.^30^ The alternative RAS signaling is mainly represented by the Ang (1–7)/MAS receptor signaling pathway. Its functions have been recently studied, mainly in cardiovascular regulation.^31^ Interestingly, it has been reported that Ang (1–7) also plays a role in cancer. However, the effect of alternative RAS signaling in cancer is not fully understood, it seems that it is different according to the specific cancer type.^29^ Anti‐neoplastic properties of Ang (1–7) have been suggested in lung, colon, breast and PC, while cancer‐promoting effects have been suggested in renal cell cancer.^29^

RAS inhibitors (RASIs), represented by ACEIs and ARBs, are agents commonly used in the treatment of arterial hypertension, myocardial infarction, heart failure and chronic kidney disease. After being used for decades in various cardiovascular diseases, RASIs have recently received a considerable attention also in oncology.

The results of previously conducted experimental studies found that the administration of RASI resulted in decreased proliferation, motility, angiogenesis and tumor growth and increased apoptosis.^32^, ^33^, ^34^, ^35^, ^36^ Although, several anticancer effects of RASI have been suggested in experimental models, their impact on the clinical outcome of PC patients has not been fully elucidated. The impact of RASIs on favorable prognosis of patients with localized‐stage PC has been suggested by Santala et al. Their study including 14,422 patients undergoing surgery showed that the use of ARBs was associated with decreased risk of cancer‐specific death.^19^ Similar findings have been reported by Siltari et al. who observed an association between the use of ARBs and improved cancer‐specific survival in a heterogeneous cohort of 8253 PC patients treated with ADT (HR = 0.81, 95% CI 0.67–0.99).^20^ Interestingly, in both studies, the positive effect seemed to be apparent particularly for ARBs, not for ACEIs, which is contradictory to our results showing longer survival in ACEIs users, and not in those using ARBs. Although the reason for such contradictory results is not clear, different patient population and treatment should be pointed out. The study by Santala et al. focused in early‐stage HSPC patients treated surgically and the study by Siltari et al. focused on HSPC patients treated with ADT.^19^, ^20^ Thus, both studies included patient cohorts different to our study focusing on mCRPC, which has substantially different biological properties compared to HSPC.^36^ Regarding the data on ACEIs, Ronquist et al. reported that the addition of captopril tend to lower recurrence rate after radical prostatectomy in a small sample prospective study.^22^ In the field of mCRPC, data on the impact of concomitant RASI use are very limited. Uemura et al. reported a promising effect of ARB, candesartan, in a cohort of heavily pretreated mCRPC patients in a Phase II pilot trial.^23^ The data from a retrospective study, recently reported by Wilk et al., including 93 mCRPC men treated with ABI in the post‐chemotherapy setting show longer time to treatment failure in RASI users versus nonusers (HR = 0.61, 95% CI 0.4–0.94, *p* = 0.02), while the type of RASI was not specified.^24^ It is worth mentioning that their study was substantially limited by the small number of patients included (in total: 93, 37 [40%] used RASIs), which resulted in the absence of relevant assessments of OS and of the specific role of RASI subgroups (i.e., ARBs vs. ACEIs). Similarly, we found significantly better outcome for mCRPC patients using RASIs in general. Furthermore, among all RASIs included in our study, the impact on survival was significant only for ACEIs. As the different effects of ACEIs and ARBs cannot be clearly explained, this issue should be further examined. However, we can hypothesize that the beneficial effect of ACEIs could be predominantly based on their influence on the alternative RAS pathway, besides the inhibition of Ang II formation preventing downstream signaling mediated by AT1R. It has been proposed that ACEIs do not interfere with the conversion of Ang I to Ang (1–9) catalyzed by endopeptidases. After the conversion, Ang (1–9) is cleaved by ACE‐2, which is not affected by ACEIs, to become Ang (1–7) binding to MAS receptors.^37^, ^38^ The anticancer effects of Ang (1–7)/MAS signaling have been mentioned above. Noatably, it has been demonstrated that Ang (1–7) is able to effectively reduce prostate cancer metastasis in experimental models with androgen‐independent prostate cancer cells.^39^ Aditionally, when the effect of ACEIs was analyzed for ENZ and ABI users separately, both groups showed longer rPFS and OS of ACEI users, while the statistical significance was reduced (showing a significant result only for rPFS in ENZ users) in comparison to the effect observed in the whole sample. This is, however, to be expected as the statistical sample got smaller with the separation of ENZ and ABI users. The fact that both groups showed similarly reduced significance could be an indication that both ENZ and ABI users contributed to the effect of ACEIs observed in the whole sample to a similar degree.

BBs represent another commonly used antihypertensive medication with various anticancer properties previously suggested in experimental studies.^40^, ^41^, ^42^ However, their prognostic role in PC patients is unclear and the findings obtained from mostly retrospective studies are inconsistent.^14^, ^15^, ^16^, ^17^, ^18^, ^19^, ^20^, ^21^, ^24^ We did not find a significant impact of concomitant use of BBs on the survival of mCRPC patients in our study. Our results are in agreement with several studies assessing the role of concomitant medication in PC patients discussed above, including the study by Wilk et al. focused on mCRPC patients.^14^, ^19^, ^20^, ^24^

Major limitations of the present study represent the retrospective design and the limited number of patients, in particular those using a specific concomitant antihypertensive medication. The dosage and duration of the observed concomitant medication was not available. Furthermore, the present study did not include any control cohort of patients treated with other type systemic therapy (e.g., chemotherapy), thus it cannot be clearly interpreted that the concomitant use of ACEIs impacts specifically the efficacy of NHTs or whether it could improve the prognosis of mCRPC patients in general. Another potential limitation of our study is that there were included patients treated with two NHTs with different mechanism of action represented by ENZ and ABI and the specific molecular mechanism of the additive effect of ACEIs cannot be clearly explained. Nevertheless, according to our best knowledge, the present study is the largest one published so far focusing on the potential impact of the concomitant use of antihypertensive medication on survival of patients with mCRPC receiving NHTs. Moreover, the strength of this study is that all the patients were treated in a single clinical Center under similar conditions of common clinical practice.

## 5 CONCLUSIONS

The results of our retrospective study suggest a positive impact of the concomitant use of ACEIs on outcome of mCRPC patients receiving ENZ or ABI. Aditionally, our findings indicate that ACEIs could represent a preferred type of antihypertensives for mCRPC patients considered for the treatment with NHTs. The effect of combination of RASIs, particularly ACEIs, with NHTs in mCRPC patients might be further studied.

## AUTHOR CONTRIBUTIONS

**Ondřej Fiala:** Conceptualization (lead); data curation (lead); investigation (lead); methodology (lead); project administration (lead); writing – original draft (lead). **Petr Hošek:** Data curation (equal); formal analysis (lead); methodology (equal); writing – original draft (equal). **Hana Korunková:** Investigation (equal). **Milan Hora:** Investigation (equal); writing – review and editing (equal). **Jiří Kolář:** Investigation (equal). **Ondřej Šorejs:** Investigation (equal). **Ondřej Topolčan:** Writing – review and editing (equal). **Jan Filipovský:** Writing – review and editing (equal). **Václav Liška:** Writing – review and editing (equal). **Matteo Santoni:** Writing – review and editing (equal). **Sebastiano Buti:** Writing – review and editing (equal). **Jindřich Fínek:** Writing – review and editing (equal).

## FUNDING INFORMATION

This study was supported by the Charles University Research Fund (Cooperatio No. 43—Surgical Disciplines), the Institutional Research Fund of University Hospital Pilsen, FN 00669806 and by the European Union's Horizon 2020 research and innovation programme under grant agreement N856620 and by the project National Institute for Cancer Research—NICR (Programme EXCELES, ID Project No. LX22NPO5102)—Funded by the European Union—Next Generation EU.

## CONFLICT OF INTEREST STATEMENT

OF received honoraria from Novartis, Janssen, Merck and Pfizer for consultations and lectures unrelated to this project. Jindrich Finek has received honoraria from Astra Zeneca, Roche, and Novartis for consultations and lectures unrelated to this project. Jan Filipovsky has received honoraria from Servier a ProMED.CS, all unrelated to this project. MS has received research support and honoraria from Janssen, Bristol Myers Squibb, Ipsen, MSD, Astellas and Bayer, all unrelated to this project. SB received honoraria as speaker at scientific events and advisory role by BMS, Pfizer, MSD, Ipsen, AstraZeneca, Merck, all unrelated to this project. PH, HK, MH, JK, OS, OT and VL declare that they have no conflicts of interest that might be relevant to the contents of this manuscript.

## Supporting information


Data S1:

## Data Availability

The datasets generated and/or analyzed during the current study are not publicly available due to patient data security but are available from the corresponding author on reasonable request.
