# Supplementary material for: Concomitant antihypertensive medication and outcome of patients with metastatic castration‐resistant prostate cancer receiving enzalutamide or abiraterone acetate
Source: Cancer Med. 2024 Jan 2;13(1):e6853. doi: 10.1002/cam4.6853 (PMC10807645; doi:10.1002/cam4.6853)
Supplement: Supplementary file 1 — Data S1: [file CAM4-13-e6853-s001.docx]

**Supplementary data**

**Table 1S:** The Kaplan-Meier analysis with Gehan-Wilcoxon test assessing the impact of the selected concomitant medication on radiographic progression-free survival (rPFS) and overall survival (OS), analyzed separately for abiraterone acetate and enzalutamide.

| **Medication** | **rPFS** | |  | **OS** | |
| --- | --- | --- | --- | --- | --- |
|  | **Median survival months, (95% CI)** | ***p* value** |  | **Median survival months, (95% CI)** | ***p* value** |
| **Abiraterone only** | | | | | |
| **Angiotensin-converting enzyme inhibitors (ACEIs)** |  | 0.0728 |  |  | 0.0692 |
| Non-users | 11.7 (10.1–14.7) |  |  | 22.7 (18.8–33.8) |  |
| Users | 21.1 (11.5–28.9) |  |  | 31.0 (not available) |  |
| **Angiotensin II receptor blockers (ARBs)** |  | 0.4879 |  |  | 0.3547 |
| Non-users | 12.7 (10.6–16.2) |  |  | 22.8 (19.8–32.8) |  |
| Users | 14.8 (10.1–29.3) |  |  | 31.9 (not available) |  |
| **Renin-angiotensin system inhibitors (RASIs) = ACEIs or ARBs** |  | 0.0661 |  |  | 0.0378 |
| Non-users | 11.4 (9.7–14.4) |  |  | 22.2 (18.2–31.4) |  |
| Users | 19.0 (11.5–23.4) |  |  | 31.5 (not available) |  |
| **Enzalutamide only** | | | | | |
| **Angiotensin-converting enzyme inhibitors (ACEIs)** |  | 0.0224 |  |  | 0.1705 |
| Non-users | 9.4 (7.8–12.7) |  |  | 24.6 (19.7–32.6) |  |
| Users | 12.5 (9.8–17.1) |  |  | 33.1 (20.1–40.7) |  |
| **Angiotensin II receptor blockers (ARBs)** |  | 0.8039 |  |  | 0.7780 |
| Non-users | 10.0 (9.1–12.7) |  |  | 27.1 (19.7–36.2) |  |
| Users | 10.6 (7.8–20.6) |  |  | 27.5 (15.8–44.4) |  |
| **Renin-angiotensin system inhibitors (RASIs) = ACEIs or ARBs** |  | 0.0269 |  |  | 0.3766 |
| Non-users | 9.0 (6.9–12.6) |  |  | 23.1 (15.9–33.7) |  |
| Users | 12.6 (9.7–17.9) |  |  | 31.0 (22.7–38.4) |  |


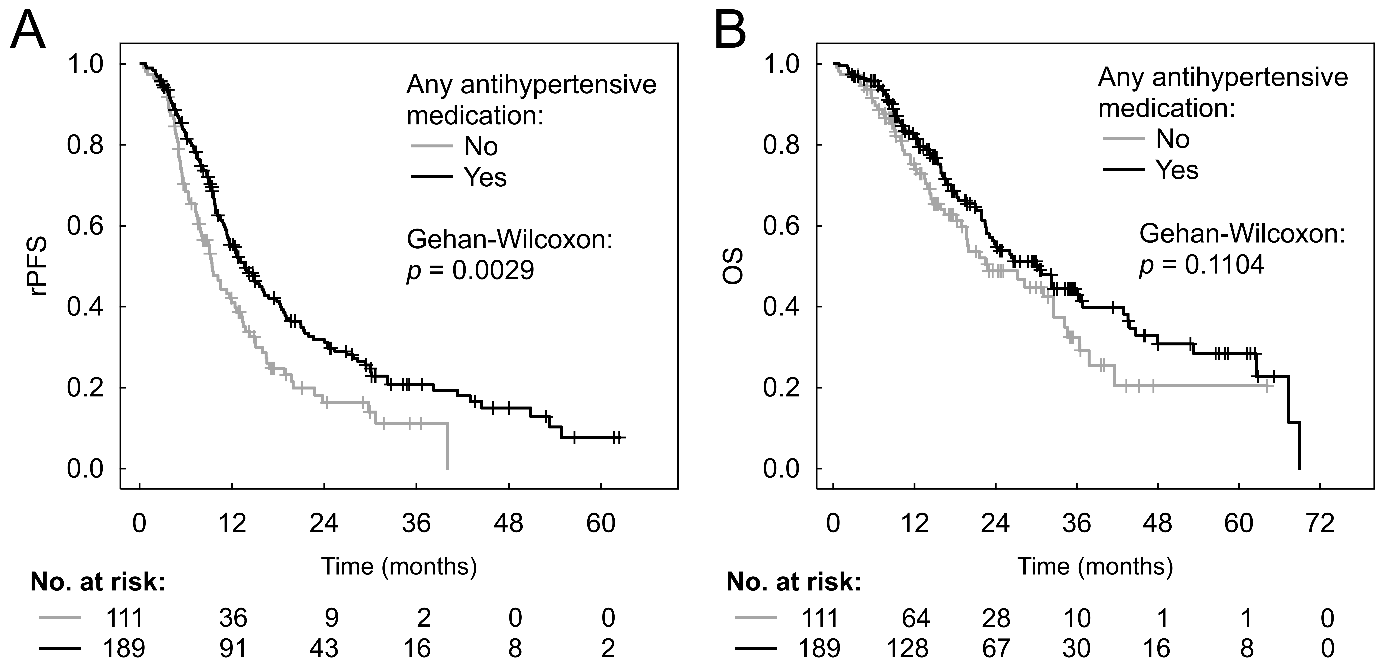


**Figure 1S**. Kaplan-Meier estimates of radiographic progression-free survival (rPFS) (A) and overall survival (OS) (B) according to the use any antihypertensive medication.
